# In Vivo Kinematic Analysis of the Axial Shoulder Rotation in the Standing and Supine Positions Using 3D/2D Registration and Electromyography

**DOI:** 10.7759/cureus.46154

**Published:** 2023-09-28

**Authors:** Tomonori Kenmoku, Keisuke Matsuki, Masaru Sonoda, Takumi Ishida, Shuichi Sasaki, Yu Sasaki, Ryo Tazawa, Scott A Banks, Masashi Takaso

**Affiliations:** 1 Department of Orthopaedic Surgery, Kitasato University Hospital, Sagamihara, JPN; 2 Sports Mecine & Joint Center, Funabashi Orthopaedic Hospital, Funabashi, JPN; 3 Division of Radiology, Seirei Sakura Citizen Hospital, Sakura, JPN; 4 Division of Rehabilitation, Kitasato University Hospital, Sagamihara, JPN; 5 Sports Medicine & Joint Center, Funabashi Orthopaedic Hospital, Funabashi, JPN; 6 Department of Orthopaedic Surgery, Kitasato University School of Medicine, Sagamihara, JPN; 7 Department of Mechanical & Aerospace Engineering, University of Florida, Gainesville, USA

**Keywords:** posture, muscle activity, 3d/2d registration techniques, acromiohumeral distance, scapula motion, shoulder rotation

## Abstract

Background

There has been no report comparing shoulder kinematics and muscle activities during axial shoulder rotation in different positions. The purpose of this study was to investigate differences in shoulder kinematics and muscle activities during axial shoulder rotation in healthy subjects between standing and supine positions using three-dimensional/two-dimensional (3D/2D) registration techniques and electromyography (EMG).

Methods

Eleven healthy males agreed to participate in this study. We recorded the fluoroscopy time during active shoulder axial rotation with a 90° elbow flexion in both standing and supine positions, simultaneously recording surface EMG of the infraspinatus, anterior deltoid, posterior deltoid, and biceps brachii. Three-dimensional bone models were created from CT images, and shoulder kinematics were analyzed using 3D/2D registration techniques. Muscle activities were evaluated as a ratio of mean electromyographic values to 5-sec maximum voluntary isometric contractions.

Results

Scapular kinematics during axial shoulder rotation in the supine position showed similar patterns with those in the standing position. The scapula was more posteriorly tilted and more downwardly rotated in the supine posture than in standing (P < 0.001 for both). Acromiohumeral distance (AHD) in the supine posture was significantly larger than in standing. Muscle activities showed no significant differences between postures except for biceps (P < 0.001).

Discussion

Shoulder kinematics and muscle activities during axial rotation were similar in pattern between standing and supine postures, but there were shifts in scapular pose and AHD. The findings of this study suggest that posture may be an important consideration for the prescription of optimal shoulder therapy following surgery or for the treatment of shoulder disorders.

## Introduction

The shoulder has the widest range of motion in all human joints, and a variety of muscles are involved in complex shoulder motions. The rotator cuff plays an especially important stabilizing role by centering the humeral head in the glenoid during shoulder motion [1-3]. Glenohumeral (GH) internal/external rotation is involved in various shoulder movements [4,5], and many authors have reported on shoulder kinematics and muscle activities during axial shoulder rotation [6-9]. Some studies, however, examined rotations during standing or sitting, while others were performed with subjects in the supine position. Since the activities of shoulder muscles are influenced by body posture [9], we cannot know if shoulder muscle activities or kinematics examined in different positions are comparable. In addition, cuff exercise is indicated for various shoulder disorders and is usually performed in the standing or sitting position [7,9-15], but there has been no reported evidence that these are the best positions to maximize exercise effects. The GH joint, especially the scapula, is considered susceptible to postural changes because of the pressure between the floor and the trunk due to the supine posture. As a result, the output of rotator cuff muscles and other muscles during rotator cuff function training may be altered. In addition, the acromiohumeral distance (AHD) would also be influenced by the posture. To the best of our knowledge, there has been no report comparing shoulder kinematics and muscle activities during axial shoulder rotation between different body postures.

Three-dimensional/two-dimensional (3D/2D) registration techniques can provide in vivo kinematic measurements with good accuracy [16-18]. In these techniques, the 3D kinematics of bones or implants are determined by registering 3D models to the silhouette of bones or implants on fluoroscopic images [16-18]. 3D/2D registration techniques were originally developed to examine total knee arthroplasty kinematics, but they have also been used extensively for shoulder kinematic studies [6,18-22]. Muscle activities during joint motion can be simultaneously examined by recording electromyography (EMG) during fluoroscopy.

The purpose of this study was to investigate differences in shoulder kinematics and muscle activities during axial shoulder rotation in healthy subjects between standing and supine body postures using 3D/2D registration techniques and EMG. We hypothesized that the shoulder kinematics and muscle activities would be influenced by the posture. Specifically, scapular motion and muscle activities would be altered in the supine position by being pressed against the table, and AHD would be smaller in the supine position due to its reduced influence of gravity.

## Materials and methods

Participants

All procedures in this study were performed in accordance with the ethical standards of our institutional research committee and with the Helsinki Declaration. The experimental protocol was conducted in accordance with the guidelines of our local ethics organization for clinical research. The protocol of this study was approved by the institutional review board of our institute, and informed consent was obtained from all participants. Eleven healthy males who had no previous shoulder injuries or disorders agreed to participate in this study. The demographics of the participants were as follows: age, 29 ± 7 years; height, 171 ± 6 cm; body weight, 67 ± 7 kg; and body mass index, 22.9 ± 2.7. All subjects were right-handed.

Experimental protocol

Fluoroscopic images of shoulder axial rotation with the arm at the side were recorded using a flat panel director (FPD; DREX-WIN 64/12, Toshiba, Otawara, Japan). The frame rate was set at 15 frames/sec. We recorded shoulder axial rotation with a 90° elbow flexion in both standing and supine positions. Subjects stood or laid down with their back on the table, and active shoulder rotation at the side was performed with a 90° elbow flexion in 10 sec from maximum internal rotation to maximum external rotation and then to maximum internal rotation in 10 sec.

**Figure 1:**
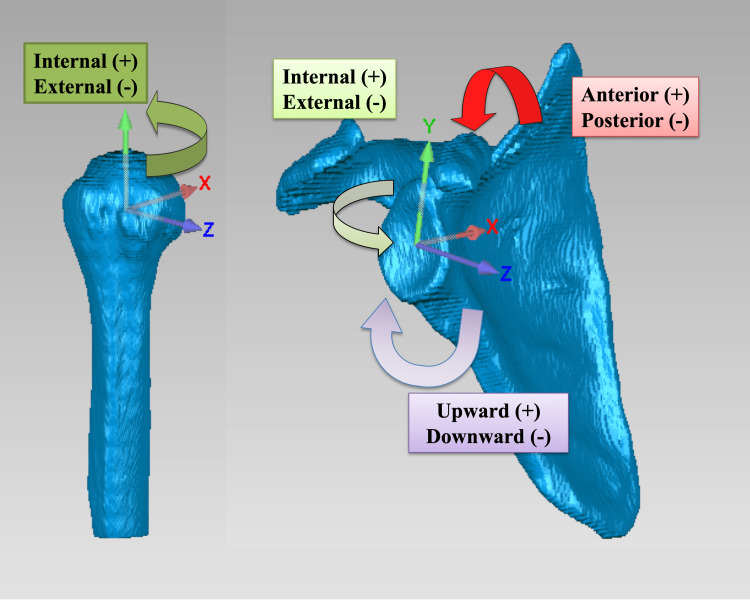
Anatomic coordination systems of the proximal humerus and scapula (right shoulder) Internal/external rotation of the humerus was defined as rotation about the y-axis The motions of the scapula were defined as scapula tilts about the x-axis, internal/external rotation about the y-axis, and upward/downward rotation about the z-axis

Each subject underwent computed tomography (CT) of the shoulder with a 512 × 512 image matrix, a 0.625 × 0.625 pixels dim, and a 1.25 mm thickness (Lightspeed 16, GE Medical Systems, Milwaukee, WI). The CT images were segmented, and 3D surface models of the proximal humerus and the scapula were created using ITK-snap software (Penn Image Computing and Science Laboratory, Philadelphia, PA) [23]. Anatomic coordinate systems were embedded in each model using Geomagic Studio (3D Systems, Rock Hill, SC) (Figure 1).

The humeral origin was set at the centroid of the humeral head. The y-axis was parallel to the humeral shaft. The anatomical neck plane was defined, and the line formed by the neck plane and the plane perpendicular to the y-axis was defined as the z-axis. The x-axis was perpendicular to both the y- and z-axes. The scapula origin was defined as the midpoint of the line connecting the superior and inferior glenoid poles, and the y- and z-axes were pointed superiorly and anteriorly, respectively, according to reported conventions [18].

Surface EMG of the infraspinatus (ISp), anterior deltoid (AD), posterior deltoid (PD), and biceps brachii (Bii) was simultaneously recorded during the rotation activity (BioLog DL-4000, DKH Co., Tokyo, Japan). Surface electrodes (DL-141, DKH Co., Tokyo, Japan) were placed over the belly of each muscle (20 mm inter-electrode distance) according to the Perotto method [24]. Muscle activities were recorded at a frequency bandwidth of 5-500 Hz and a sampling frequency of 1000 Hz. The EMG signals had an active lead amplification of 400× with a 5-500 Hz (−3 dB) band pass filter, a common mode rejection ratio (CMRR) of >110 dB, and an input impedance of >200 MΩ. Data were full-wave rectified and filtered (m-Scope2, DKH Co., Tokyo, Japan) with a 100 msec moving average to create a linear envelope. Maximum voluntary contraction (MVC) value of each muscle during a 5-sec isometric contraction was measured using a hand-held dynamometer (μTas F-1, Anima Corp., Tokyo, Japan), and muscle activities were evaluated as a ratio of mean EMG values to MVC (%MVC) as following established techniques [25].

Model-image registration

**Figure 2:**
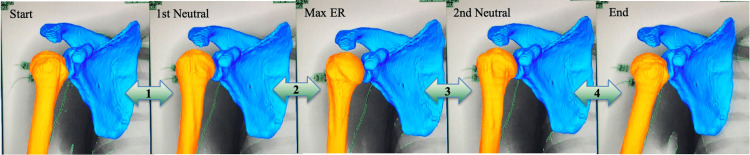
Measurement points and phases (right shoulder) We divided rotational motion into the following five points: starting point (Start), the neutral point during external rotation (1st Neut), maximum external rotation (Max ER), the neutral point during internal rotation (2nd Neut), and the endpoint (End) Muscle activities were evaluated during phases between each measurement point (green arrow)

Three-dimensional positions and orientations of the humerus and scapula were determined using 3D/2D model-image registration techniques (Figure 2) [19-22].

The accuracy of these techniques using single-plane images was 0.47 mm for in-plane translation, 1.53 mm for out-of-plane translation, 0.76° for in-plane rotation, and 3.72° for out-of-plane rotation in a previous study conducted in a similar manner [18].

Data processing

Cardan angles (Z-X-Y order) were used to compute the kinematics of the humerus and scapula in relation to the X-ray coordinate system and humerus kinematics relative to the scapula [26]. Axial rotation of the humerus was defined as rotation about the y-axis. Scapula motion was defined as anterior/posterior tilt about the x-axis, internal/external rotation about the y-axis, and upward/downward rotation about the z-axis. GH internal/external rotation was defined as a rotation of the humeral y-axis relative to the scapula coordinate system, and 0° of rotation was defined as the neutral position. Superior/inferior humeral translation was defined as the motion of the humeral origin relative to the scapular origin along the scapular y-axis. AHD was computed as the closest distance between the humeral head and acromion.

The continuous motion from beginning to end was divided into five time points as the arm moved from maximum internal rotational position (Start) to maximum external rotational position (Max ER) and then to maximum internal rotational position (End). The neutral positions during external and internal rotation were named 1st and 2nd neutral positions, respectively. In addition, muscle activities were evaluated as %MVC during phases between each measurement point (Figure 2).

Statistic analysis 

All statistical analyses were performed using JMP Pro software, version 14.1 (SAS Institute Inc., Cary, NC, USA). Results are presented as the mean ± standard deviation (SD). All data were confirmed as a normal distribution using the Shapiro-Wilk test (P > 0.05).

The Mann-Whitney U-test was used for the comparison of GH rotation angles at Start, Max ER, and End. The two-factor (posture and measurement point) repeated-measures analysis of variance (ANOVA) was used for comparison of kinematic and EMG data. When the ANOVA found a significant difference between the postures, the Mann-Whitney U-test was performed at each point as a post hoc test. When there was a significance in the factor of measurement point, the Steel-Dwass test was performed for comparison among measurement points in each position. Statistical analyses of kinematics during external and internal rotations were performed separately. For all statistical analyses, the significance was defined as P < 0.05.

## Results

Glenohumeral kinematics

Every participant had no deformity in the GH joint in the CT. In the standing and supine positions, the mean IR angles at Start were 45° ± 15° and 48° ± 13°, respectively; the mean ER angles at Max ER were 51° ± 18° and 45° ± 14°, respectively; and the mean IR angles at End were 50° ± 11° and 57° ± 14°, respectively. There were no significant differences at all measured points between the standing and supine positions (P = 0.47, 0.45, and 0.16, respectively).

**Figure 3:**
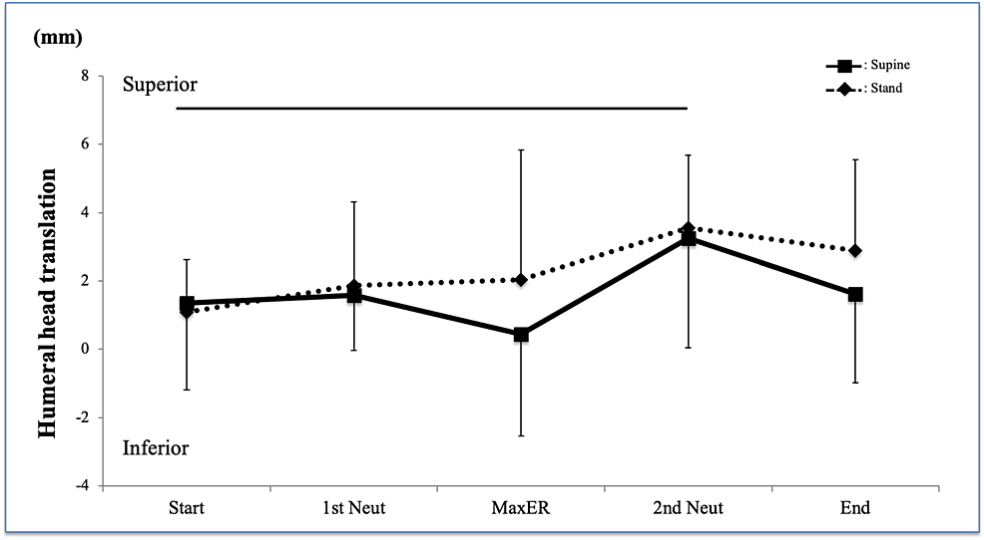
Superior/inferior translation of the humerus There was no significant difference between the postures Horizontal line = significant difference compared to Max ER ER, external rotation; IR, internal rotation; Neut, neutral position

Superior/inferior translation of the humeral head in both postures showed a similar pattern except at Max ER, and there were no interactions (Figure 3, P = 0.72). There was no significant difference between the postures (P = 0.19). The changes in superior/inferior translation during the rotational motion were significant (P = 0.038), and the Steel-Dwass test in each position revealed that there was a significant difference between the Start and 2nd neutral positions (P = 0.022).

Scapular kinematics

**Figure 4:**
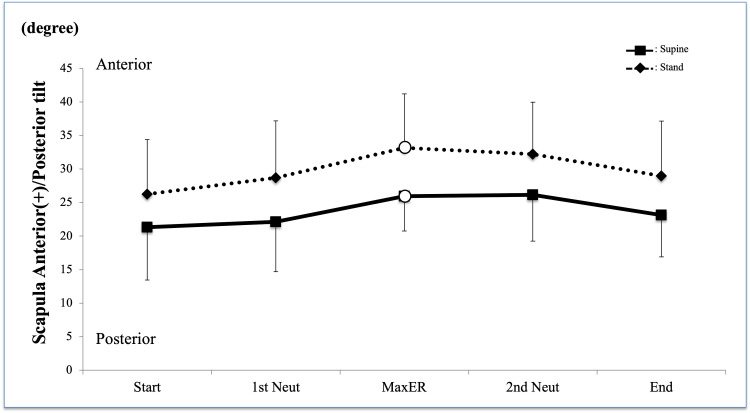
Scapula kinematics anterior/posterior (A/P) tilt There was a significant difference between the standing and supine positions (P < 0.001); however, a similar kinematic pattern with approximately a 6° offset was observed between the positions White circle = significant difference between the standing and supine positions ER, external rotation; IR, internal rotation

Scapular tilt patterns in standing and supine postures were similar but showed approximately a 6° offset throughout the activity (Figure 4). Two-factor ANOVA revealed no interactions between scapular tilt (P = 0.99). A significant difference was confirmed between the postures (P < 0.001), and the post hoc test showed a significant difference between the postures at Max ER (P = 0.020). However, there was no significant difference in the change of scapular tilt during rotational motion (P = 0.055). 

**Figure 5:**
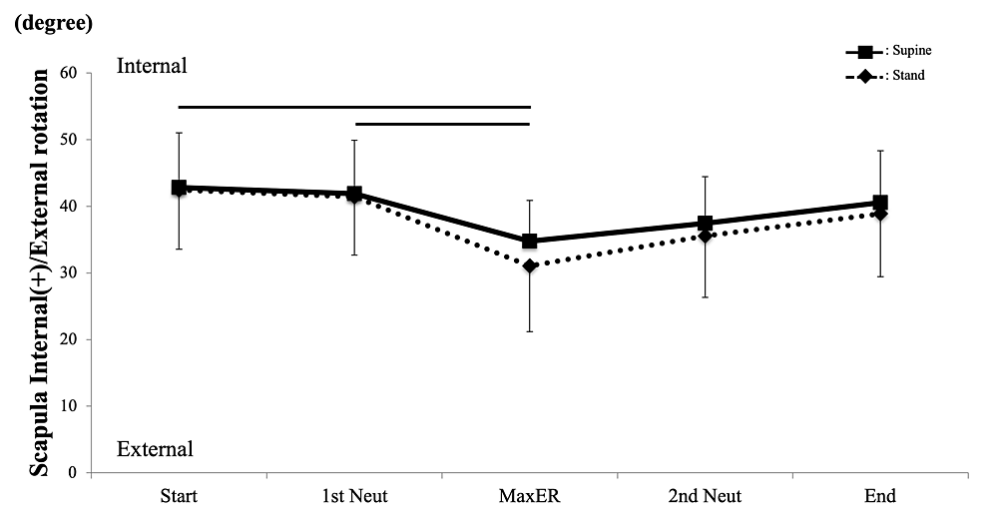
Scapula kinematics internal/external (I/E) rotation There was no significant difference between the two positions Horizontal line = significant difference compared to Max ER

Scapula internal/external rotation showed a similar kinematic pattern (Figure 5), and there were no interactions between scapular rotations (P = 0.97). There was also no significant difference between the postures (P = 0.32). Significant changes during rotational motion were detected (P = 0.001). The post hoc test revealed that the angle at Max ER was significantly smaller than those at Start and 1st Neutral in standing (P = 0.046 and 0.046, respectively); however, no significance was found in the supine position. 

**Figure 6:**
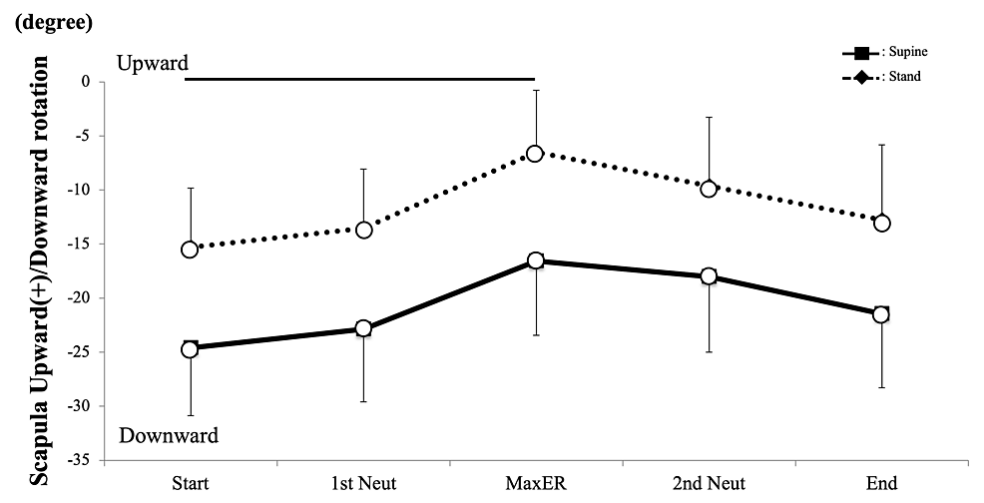
Scapula kinematics upward/downward (U/D) rotation The kinematic patterns in the standing and supine positions were parallel throughout the activity with approximately a 9° of separation (P < 0.001) White circle = significant difference between the standing and supine positions ER, external rotation

The kinematic patterns of upward/downward rotation in the standing and supine positions were parallel throughout the activity with an approximately 9° offset (Figure 6), and no interactions were revealed between scapular rotations (P = 0.99). The upward rotation angle in the standing position was significantly larger than that in the supine position (P < 0.001 for both). The post hoc test revealed that there were significant differences at all measured points (P = 0.004, 0.004, 0.010, 0.014, and 0.010). Significant changes during rotational motion were detected (P < 0.001), and scapular upward rotation at Max ER was significantly larger than those at Start and End (P = 0.040 and 0.029, respectively).

Acromiohumeral distance

**Figure 7:**
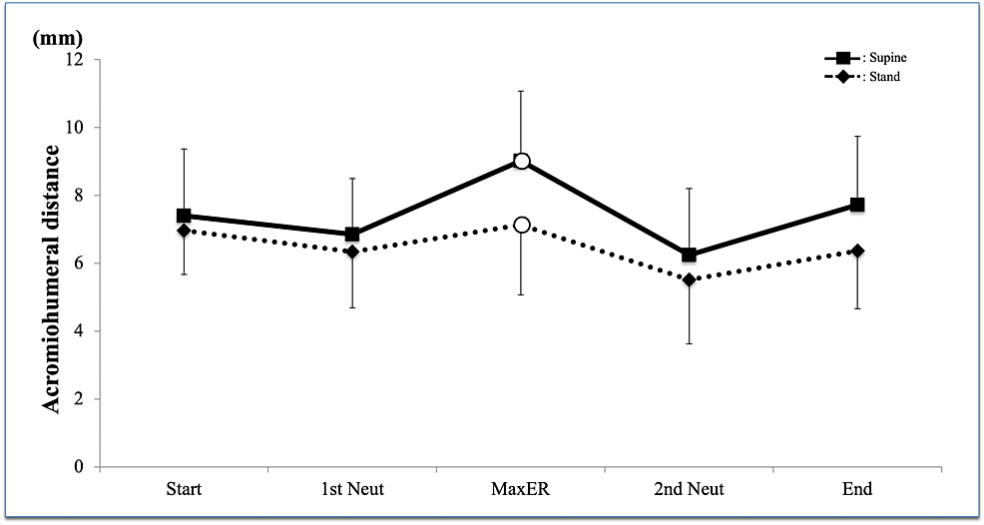
Acromiohumeral distance Acromiohumeral distance in the supine position was significantly larger than that in standing (P = 0.005) The post hoc test revealed that there was a significant difference at Max ER (P = 0.02) White circle = significant difference between the standing and supine positions ER, external rotation

The mean AHD was larger in the supine position throughout the activity but showed a similar trend in both postures with the largest value at Max ER and the smallest at 2nd neutral (Figure 7). The two-way ANOVA of AHD also revealed no interaction between rotation directions (P = 0.72). AHD in the supine position was significantly larger than that in the standing position during rotational motion (P = 0.008); however, the post hoc test found no significant difference in every point. The change of AHD was significant during IR motion (P = 0.006), and the post hoc test detected that AHD at Max ER was significantly larger than that at 2nd Neutral (P = 0.025).

Muscle activities 

Table 1 shows the muscle EMG during rotation. There were only a few differences in muscle activation between body postures. There was no significant difference in ISp EMG between the supine and standing positions, although ISp EMG was significantly increased at the terminal external rotation regardless of postures. On the other hand, PD in the supine position gradually increased during external rotation, although that in the standing was increased significantly at the terminal external rotation. AD EMG was more or less active during different phases of activity without a significant difference in posture. Bii were more active when supporting the arm against gravity at the Start point; however, no significant difference was found regardless of posture.

**Table 1:** Electromyography results Phases: 1, between Start and 1st Neutral; 2, between 1st Neutral and Max ER; 3, between Max ER and 2nd Neutral; 4, between 2nd Neutral and End ^†^The P-value between the supine and stand postures was analyzed at each point using the Mann-Whitney U-test ^††^The P-value among rotational motion phases was analyzed using the Mann-Whitney U-test ISp, infraspinatus; AD, anterior deltoid; PD, posterior deltoid; Bii, biceps brachii

		External rotation			Internal rotation		
Phase		1	2	P-value^††^	3	4	P-value^††^
ISp	Supine	6.1 ± 3.4	12.0 ± 8.9	0.048	11.8 ± 5.4	4.8 ± 2.4	0.002
	Stand	5.5 ± 3.1	17.3 ± 10.0	0.003	15.4 ± 14.1	7.4 ± 8.3	0.014
	P-value^†^	0.62	0.14		0.72	0.62	
AD	Supine	2.2 ± 1.5	1.8 ± 0.9	0.74	2.0 ± 1.6	2.4 ± 2.3	0.74
	Stand	1.6 ± 0.5	1.7 ± 0.5	0.32	1.8 ± 0.9	1.9 ± 1.1	0.82
	P-value^†^	0.87	0.92		0.81	0.97	
PD	Supine	2.4 ± 1.4	3.9 ± 2.8	0.15	3.4 ± 2.0	1.9 ± 0.8	0.57
	Stand	2.6 ± 1.4	5.1 ± 2.9	0.012	4.4 ± 3.6	3.0 ± 2.8	0.10
	P-value^†^	0.67	0.12		0.84	0.62	
Bii	Supine	2.7 ± 1.4	2.5 ± 1.2	0.90	2.6 ± 1.5	2.8 ± 1.6	0.65
	Stand	4.3 ± 1.7	3.8 ± 1.6	0.58	3.9 ± 1.9	4.4 ± 2.2	0.64
	P-value^†^	0.020	0.061		0.14	0.053	

## Discussion

Scapular kinematics during axial shoulder rotation showed similar rotation patterns in the supine and standing postures, but the scapula was significantly more posteriorly tilted and downwardly rotated when supine. AHD in the supine position was significantly larger than that in the standing position. Muscle activities demonstrated no significant differences between postures except for the Bii, which supports the forearm against gravity during standing. In addition, the external rotation in the supine position could facilitate ISp without a significant increase in PD EMG, unlike that in standing.

We hypothesized that shoulder kinematics would be different between postures because the scapula is pressed against the table in the supine posture. We did observe an offset in the supine scapular posture, but relative displacements during arm rotation were similar to standing. More specifically, scapular tilt and rotation are influenced by posture. Thoracic kyphosis decreased in the supine position compared to the standing position [27,28], showing a concomitant posterior scapular tilt [29]. With standing, we suspect postural adjustments against gravity would include increased activity of the upper trapezius to rotate the scapula upwardly. Indeed, several studies have reported decreased upper trapezius activity during supine shoulder motion compared to standing [11,30]. Unfortunately, we did not examine the upper trapezius activity with EMG in this study and require further investigation to prove this assumption. 

Contrary to our expectation, AHD in the supine position was larger than that in the standing position. Several studies have reported that shoulders with subacromial impingement syndrome show decreased scapular posterior tilting compared to healthy shoulders, which may be associated with decreased AHD [31,32]. Considering these findings, the significant posterior tilt of the scapula might contribute to the larger supine AHD. There was no significant difference in the humeral head superior/inferior translation between the two positions, further supporting the idea that larger supine AHD results from more posterior scapular tilt. 

Our results showed no significant differences in muscle activity between the two positions except for the biceps. This finding is similar to a previous report that biceps activity was significantly higher in a seated position than in the supine position during isometric shoulder ER with the arm at the side [11]. The biceps activity should be higher in upright positions to maintain the elbow at a 90° flexion against gravity, while the activity should be lower in the supine position because gravity should not induce a flexion torque during IE rotation with the elbow at a 90° flexion. There was no significant difference in ISp or PD activity between the two positions, and muscle activity was higher at the end of external rotation. Consistent with our results, Heuberer et al. reported that ISp activity increased with the degree of external rotation during axial shoulder rotation with the arm at the side [33]. Similarly, other studies have reported that PD activation was similar to ISp activation in isometric external rotation [9,11,34].

Active external rotation of the shoulder at the side in the supine position showed greater AHD with smaller biceps activity than in the standing position, while ISp activity was not different between the two positions. These results imply that ISp exercise in the supine position might be more beneficial than that in the standing position because of the lower risk of subacromial impingement and smaller influence of biceps activity. Further clinical studies are required to prove this hypothesis regarding the effectiveness of ISp exercise in the supine position.

Limitations

The major limitation was the small number of subjects, single-sex, and narrow age range. We were required to limit subjects because of radiation exposure. Second, we only assessed the activity of four muscles. Other muscles, such as the lateral deltoid, trapezius, or subscapularis, may have some influence on shoulder kinematics [11,14,35,36]. Third, we used surface EMG, which could be influenced by positioning and crosstalk artifacts. The use of fine-wire electrodes enables selective evaluation of muscle activities and should be considered for future similar studies.

## Conclusions

GH and scapular kinematics during axial shoulder rotation were similar between the standing and supine positions, but the supine scapula was more posteriorly tilted and downwardly rotated. AHD was larger in the supine position than in the standing position. Muscle activities showed no significant differences between postures except the biceps. Our findings suggest that shoulder kinematics and muscle activities in the standing and supine postures are largely similar, but postural shifts in the supine scapula may provide larger AHD with resulting advantages for rehabilitation of shoulders with impingement.
